# Hospital Appeals and Hospital Abuse

**Published:** 1906-07-07

**Authors:** 


### Editor's Letter-Box.

Our Correspondents are reminded that prolixity is a great bar to publication, and that brevity of style and conciseness of statement greatly facilitate early insertion.]

### HOSPITAL APPEALS AND HOSPITAL ABUSE.

Mr. Charles Corfield, M.R.C.S., L.R.C.P., writes to us: In individual hospital appeals by their respective committees there is, one might say with perfect truth, quite a competition to record the greatest number of patients treated as an argument for further help. If, however, another picture were drawn in the pulpit or the Press of daily hospital abuse, and the public learned that their well-meant charity was in part expended in affording relief to those far removed even from the term of poverty, and that in a large percentage of these multi-figured statistics the cases could not even be called deserving, then I think the donors would be more chary of their charity, or would at least demand that more stringency be exercised in its expenditure. It may be urged that printed regulations and notices regarding fitness for hospital treatment are put up in all parts of such institutions; this, of course, is a necessary formality, but by itself is about as efficacious in stopping abuse as the printed notices of the railway companies forbidding gratuities to their employés is in stopping tips. It is necessary for hospital committees not only to formulate such bye-laws, but to see that they are carried out as well. I believe the real cause of so much latitude in admitting patients to treatment is as I have above indicated—that most hospitals make no serious attempt to grapple with this evil because impressive statistics are their chief claim to the bounty of the charitable public.

And so it comes about that the increase in patients with the necessary increase of expenditure seems to be a matter for congratulation rather than regret.

As a late hospital resident I have no hesitation in saying that nearly half the cases admitted medically to the out-patient departments could well afford to attend a medical man in private practice. Their ailments are mainly gastro-intestinal troubles, largely dependent on wrong living, and to have to pay a fee for each medical consultation would probably act as a wholesome deterrent in such cases as these.

I am certain, too, that if a great many of these out-patients had to pay for their treatment as they pay for the other commodities in life, many of their ailments would disappear.

The facility with which out-patient treatment can now be got has contributed largely to the low fees which medical men in poorer districts have had to charge in their private practice. The sixpenny doctors, whether for good or evil, are the outcome of such hospital competition.

It is, I think, time that the subscribing public awoke to the true facts of the case. Every year the burden of hospital debt becomes heavier, and if this should go on it may be a question whether the ratepayers will not be saddled with the liabilities. Unfortunately, municipal or State aid, whenever invoked, tends to sap the springs of charity, and if it should chance that the larger portion of hospital expenditure had to come from that source it would be a tax which the heavily-burdened ratepayers would be likely to strongly resent.

Hospital abuse, then, seems to me to be an injustice; firstly, to those who support these institutions, because their contributions are mis-spent; secondly, to those who, being genuine cases of deserving poor, tend to be crowded out by others who could afford treatment elsewhere; thirdly, to general practitioners of medicine who are being robbed of their clientele by misdirected charity. I have often wondered how many of our city merchants whose merchandise is purchased by the working classes would feel if some charitable body were to manufacture and dispense their wares gratuitously. Medical attendance, whatever title it may sometimes masquerade under, is, after all, a marketable commodity too, and medical men have as much right to expect a profitable return with fair competition as the merchant or the shopkeeper.

One great difficulty in dealing with this question is, of course, that it is impossible to lay down hard and fast rules for guidance. Each case must be judged on its merits. To take an instance. Two patients come to the out-patient department of a hospital, one a man with early phthisis and needing medicine for several weeks of an expensive nature; another comes up with some stomach trouble due to over-feeding and drinking, and needing, perhaps, one bottle of medicine with the much more important directions for altering his mode of life. Though they may both be getting the same wage, the first no doubt is the more entitled to treatment. The second might well be dealt with by a private doctor. One method I might suggest would do much to mitigate the evil. Full particulars of income and family might appear on the book given to the hospital doctor, and then if the case seemed to him to want, owing to the nature of the malady, continued treatment, the out-patient book might be stamped to that effect. Other cases of a trivial nature might be seen once, and then discharged to get advice outside. Another or additional method might be to make a certificate from a qualified practitioner a *sine qua non* for hospital treatment. In both these cases an appeal, of course, would always lie to the committee or an investigating body formed for that end in view.

I may say in conclusion that for the purpose of dealing with hospital abuse there may be many different methods, but that such a reform is needed I think no one can possibly deny.

